# Heritability and correlations among learning and inhibitory control traits

**DOI:** 10.1093/beheco/araa029

**Published:** 2020-03-29

**Authors:** Ellis J G Langley, Gracie Adams, Christine E Beardsworth, Deborah A Dawson, Philippa R Laker, Jayden O van Horik, Mark A Whiteside, Alastair J Wilson, Joah R Madden

**Affiliations:** 1 Centre for Research in Animal Behaviour, College of Life and Environmental Sciences, Washington Singer Labs, University of Exeter, Exeter, UK; 2 Department of Animal and Plant Sciences, Alfred Denny Building, University of Sheffield, Western Bank, Sheffield, UK; 3 Centre for Ecology and Conservation, University of Exeter, Penryn Campus, Penryn, UK

**Keywords:** animal model, cognitive abilities, genetic correlations, general intelligence, heritability, pheasant

## Abstract

To understand the evolution of cognitive abilities, we need to understand both how selection acts upon them and their genetic (co)variance structure. Recent work suggests that there are fitness consequences for free-living individuals with particular cognitive abilities. However, our current understanding of the heritability of these abilities is restricted to domesticated species subjected to artificial selection. We investigated genetic variance for, and genetic correlations among four cognitive abilities: inhibitory control, visual and spatial discrimination, and spatial ability, measured on >450 pheasants, *Phasianus colchicus*, over four generations. Pheasants were reared in captivity but bred from adults that lived in the wild and hence, were subject to selection on survival. Pheasant chicks are precocial and were reared without parents, enabling us to standardize environmental and parental care effects. We constructed a pedigree based on 15 microsatellite loci and implemented animal models to estimate heritability. We found moderate heritabilities for discrimination learning and inhibitory control (h^2^ = 0.17–0.23) but heritability for spatial ability was low (h^2^ = 0.09). Genetic correlations among-traits were largely positive but characterized by high uncertainty and were not statistically significant. Principle component analysis of the genetic correlation matrix estimate revealed a leading component that explained 69% of the variation, broadly in line with expectations under a general intelligence model of cognition. However, this pattern was not apparent in the phenotypic correlation structure which was more consistent with a modular view of animal cognition. Our findings highlight that the expression of cognitive traits is influenced by environmental factors which masks the underlying genetic structure.

## Teaser

Cognitive traits are crucial determinants of animals’ abilities to adapt to changing environments. For cognitive traits to evolve they must be underpinned by a genetic component. Using quantitative genetics in a nondomesticated system, we show that the basic abilities to inhibit behavior and learn about visual and spatial cues are moderately heritable. This heritability, coupled with fitness consequences of individual differences now demonstrated across several species, highlights the evolutionary potential for basic cognitive abilities.

## INTRODUCTION

Understanding the genetic underpinnings of cognitive abilities provides insights into how cognitive traits are structured and have evolved. General cognitive abilities (learning, memory, executive function) underpin critical behaviors, such as foraging (Raine and Chittka 2008; Pasquier and Grüter 2016), mate choice (Shohet and Watt 2009; Araya-Salas et al. 2018), and predator avoidance (Turner et al. 2006). Importantly, performances in cognitive tasks have associated fitness consequences in wild populations (reproduction: [Ashton et al. 2018; Branch et al. 2019; Shaw et al. 2019]; survival: [Maille et al. 2016; Madden, Hall, et al. 2018; Sonnenberg et al. 2019; Langley et al. 2020]). Although this variation and the associated fitness implications are indicative of the evolutionary potential of these traits, investigation into their heritable component has received little attention in behavioral ecology. Furthermore, by exploring the genetic contribution to specific cognitive traits we can better appreciate how they are structured, that is, genetic similarity underpinning cognitive task performances (Thornton and Wilson 2015).

Heritability estimates for specific cognitive abilities are sparse (reviewed in Dukas 2004; Croston et al. 2015). Associative learning ability shows low to moderate heritability in insects (fruit flies, *Drosophila melanogaster*, h^2^ = 0.08, Lofdahl et al. 1992; honeybees, *Apis melifera capensis*, h^2^ = 0.39–0.54, Brandes 1988), whereas, in red junglefowl (*Gallus gallus*), discrimination learning, an aspect of associative learning (specifically, responding differently to two cues) showed no heritable component (h^2^ = 0.00 ± SE of 0.06, Sorato et al. 2018). Instead, in red junglefowl, genetic variation contributed to reversal learning (h^2^ = 0.25 ± SE of 0.12, Sorato et al. 2018), when rewarding and nonrewarding cues are switched. Reversal learning may require an individual to inhibit a learned behavior (Lai et al. 1995) and inhibiting a prepotent response, hereby inhibitory control, is reported to be highly heritable in humans (h^2^ = 0.99, (Friedman et al. 2008); h^2^ = 0.27–0.50, Schachar et al. 2011). Spatial learning is moderately heritable in mice (h^2^ = 0.27, Matzel et al. 2019) but has seldom been investigated in other taxa (see Croston et al. 2015). Heritability estimates have also been obtained for single factors that purport to summarize performances across batteries of cognitive tests and thus indicate a “general” intelligence. Such estimates are moderate to high in humans (h^2^ = 0.26–0.86, see Plomin and Spinath 2002) and moderate in chimpanzees (*Pan troglodytes*) (h^2^ = 0.53, Hopkins et al. 2014) and mice (*Mus musculus*) (h^2^ = 0.34–0.42, Galsworthy et al. 2005).

Much of our current understanding of the genetic contribution to cognitive abilities arises from captive bred animals that have been subject to artificial selection (reviewed in Dukas 2004; Croston et al. 2015). Direct comparison of genetic variation across traits and studies is not always easy due to differences in trait definition, statistical methodology used, and preferred standardizations of additive genetic variance (e.g., h^2^ vs. CV_A_; Houle 1992). Nevertheless, there are concerns that laboratory populations of livestock and model organisms (e.g., mice) may not be very representative of genetic variation for cognitive performance in free-living populations. For instance, reduced environmental variation in captive populations may impact genetic variance through GxE and/or levels of nongenetic variance (mice, Sauce et al. 2018). Inbreeding is also common in many captive populations, is known to influence average cognitive performance (e.g., humans, Bashi 1977; Howrigan et al. 2016; canaries (*Serinus canaria*), de Boer et al. 2016; but see *Drosophila melanogaster*, Nepoux et al. 2010), and can alter genetic variance in multiple ways (Whitlock and Fowler 1999).Thus, the existing literature arguably gives us little insight into the genetic variation that exists in populations in which cognitive traits may be under natural selection and how they may evolve. One study that measured heritability of innovative problem solving in a wild population of great tits (*Parus major*), found little support for a genetic component of variation (h^2^ = 0.04, lower credible interval ≤ 0.01, upper credible interval = 0.15, Quinn et al. 2016). However, the link between problem solving performance and cognitive ability may be convoluted, with performance in such tasks more strongly influenced by noncognitive factors such as previous experience, motivation, or persistence (van Horik and Madden 2016). Consequently, to further our understanding of genetic variation in cognitive abilities, it is desirable to measure behavior in a system that can be viewed as genetically representative of a wild population (e.g., no history of inbreeding or strong artificial selection) but in which environmental conditions at testing can be standardized across individuals.

We measured performances on four cognitive tasks (inhibitory control, visual discrimination, spatial discrimination, and spatial ability) in four generations of pheasants (*Phasianus colchicus*) and used animal models (Lynch and Walsh 1998; Wilson et al. 2010) to assess the genetic variance of each cognitive ability and investigate genetic correlations between them. We assume these broad cognitive traits represent birds’ natural foraging behavior including their ability to respond flexibly to unrewarding stimuli and learn about rewarding food locations that differ either visually or spatially. Pheasants show individual variation in inhibitory control and learning performances (Meier et al. 2017; van Horik et al. 2018; van Horik, Langley, Whiteside, Laker, et al. 2018). They show low (0.04–0.26) yet significant repeatability in individual performances across related task variants (Cauchoix et al. 2018) and their early-life cognitive performance predicts their probability of survival in the wild (Madden, Langley, et al. 2018). Their performance in such tasks is influenced by nongenetic factors including the spatial complexity of their early rearing environment (Whiteside et al. 2016) and their current and recent social environments Langley et al. 2018, 2018a, 2018b. Critically, pheasants are precocial and can be tested individually on cognitive tasks from a few weeks old, after being reared in homogenous environments without parents. This standardizes the environmental and maternal influences on variation in cognitive performances. In the United Kingdom, pheasants are reared for the first 6–8 weeks in captivity but then released into the wild during July/August where they suffer very high levels of predation and other natural hazards as well as being hunted by humans in the following autumn and winter (Madden, Hall, et al. 2018). Around 80% are dead by the start of spring, ~9 months after release, when survivors are caught up and bred from, with their eggs being hatched in incubators. Therefore, they face substantial opportunity for natural selection on traits through survival, but there is less opportunity for selection through reproductive success as choice of sexual partner is largely constrained by housing conditions.

Our current objectives are thus to ask whether, and if so to what extent, genetic variation underpins individual differences in cognitive task performances in pheasants. Furthermore, we estimate the genetic correlation structure among pairs of cognitive traits to evaluate whether genetic relationships, if present, are consistent with an underlying “general” intelligence factor. The general intelligence model posits that strong positive correlation structure will be found among different cognitive traits as performance in different tasks will reflect a single latent intelligence factor. Thus, if there is genetic variance in general intelligence we should find positive genetic correlations among traits tested (Plomin 2001; Burkart et al. 2016). In fact, previous work conducted within a single year found no evidence for a “general” intelligence factor when scrutinizing phenotypic correlations alone (van Horik, Langley, Whiteside, Laker, et al. 2018). Thus, if the previously documented absence of phenotypic relationships is reliable, and a valid proxy of genetic ones (“Cheverud’s conjecture”; Cheverud 1988; Hadfield et al. 2007) we predict no strong positive genetic correlations. However, here, we revisit this question at the level of the genotype, using a larger sample of birds assayed across multiple years.

## METHODS

This study took place over 4 years from May 2014 to July 2017 at North Wyke Research Farm, Devon, UK (50°770N, 3°90W). In the May of each year, we reared ~200 newly hatched pheasant without parents in identical housing enclosures for 9–10 weeks while we tested their individual cognitive performances (see *Chick cognitive testing*) and collected a blood sample for genotyping. Birds were individually marked and in July/August, we released them into the wild where they had access to supplementary feeding stations containing wheat and were subject to natural hazards, for example, predation and disease, but where no anthropogenic hunting took place. In the following March, prior to the birds’ first breeding season, we captured and housed surviving birds in breeding groups and collected their eggs (see *Adult breeding*) which were artificially incubated and hatched to produce the next generation. In year 1, pheasant chicks were purchased from a commercial game dealer on the day of their hatching. In years 2 and 4, the chicks were hatched from eggs collected from our captive breeding adults. In year 3, due to an incubator malfunction and low hatching success of eggs from our captive breeding adults, 80% of chicks were purchased.

### Chick cognitive testing

Chicks were housed in one of four identical housing pens (see van Horik and Madden 2016; van Horik et al. 2017 for housing details). In brief, chicks were trained to voluntarily enter the testing chamber (0.75 cm × 0.75 cm) individually. The order in which chicks enter the testing chamber, hereby *test order*, is repeatable and may reflect motivation and, or competitive ability; individuals with a lower score being more motivated/competitive and entering the chamber earlier (van Horik et al. 2017). Once in the testing chamber, an individual was presented with a freely available mealworm located in front of the testing apparatus (described below) which standardized chicks approach to the apparatus. Within a testing session, chicks had up to 2 min to interact with the testing apparatus to acquire meal worm food rewards while an experimenter recorded their behavior and operated the task apparatus (if required). Once a bird completed the task, exhibited signs of stress (flapping, pacing, or lost calling), or if 2 min had passed, the bird could leave the testing chamber and move to the outdoor area. All birds experienced two testing sessions per day, once in the morning and afternoon, Monday to Friday. In this study, we focused on four tasks that were conducted across years and which assessed either: inhibitory control, visual discrimination, spatial discrimination, and spatial learning abilities. Although we conducted a number of other tasks (see van Horik et al. 2017; Meier et al. 2017; van Horik, Langley, Whiteside, Beardsworth, et al. 2018; van Horik, Langley, Whiteside, Laker, et al. 2018), these were not included because they were not conducted across multiple years, resulting in low sample size and inadequate statistical power for estimating quantitative genetic parameters. More than 96% of participators completed all test trials, the remaining 4% completed at least half of the test trials and were included in analyses to maximize sample size (Table 1).

**Table 1:** A summary of the participation and completion rates of individuals included in animal models

Task	Years conducted	Participators *n*	100% of trials completed	70–99% of trials completed	50–70% of trials completed
Inhibitory control	1, 2, 4	341	341 = 100%	0	0
Visual discrimination	2, 3, 4	459	447 = 97%	8 = 2%	4 = 1%
Spatial discrimination	2, 3	252	234 = 93%	11 = 4%	7 = 3%
Spatial ability	2, 3, 4	456	434 = 95%	22 = 5%	0

The first column reports in which years each task was conducted. The final three columns report the number and % of individuals that completed 100%, 70–99%, or 50–70% of trials in each task.

### Inhibitory control task

We assessed inhibitory control using the detour reach paradigm in which subjects are required to retrieve a food reward from behind a transparent barrier (Boogert et al. 2011; MacLean et al. 2014; van Horik et al. 2018; Kabadayi et al. 2018). Birds were first presented with an opaque version of the task (wrapped in black tape) requiring them to learn the motor action of reaching behind the barrier to acquire food. Individuals were then given a single test session in which food was placed within a transparent version of the apparatus. Our measure of inhibitory control was the number of pecks made to the transparent barrier before retrieving the food reward in this test session. Only individuals to complete a minimum of three of four training sessions and retrieve the food in the test session were included in analyses (see Supplementary Information 1a. *Inhibitory control task* for further details).

### Learning tasks

The three remaining tasks (visual and both spatial tasks) involved foraging grids (38 cm × 14 cm × 4 cm) containing circular wells (diameter 2.8 cm), 1.2 cm apart, from which individuals could acquire mealworm rewards by pecking through crepe paper. The apparatus for discrimination tasks contained only two wells and for the spatial ability task, the apparatus contained 10 wells (see Supplementary Information 1b. *Learning tasks* for details).

### Visual discrimination

Each of the two wells was encircled by either a blue or green color cue (year 2: green was rewarded; year 3; blue was rewarded; year 4; blue was rewarded; the nonrewarded color was blocked with card). During a test trial, if the first peck was to the rewarded well it was scored as “correct” and the bird was allowed to consume the food reward before being presented with a new set of wells. If the first peck was to a blocked unrewarded well, it was scored as “incorrect” and these wells were removed and promptly replaced with a new set of wells. The location of the rewarded well was pseudorandomized between trials so that it was not in the same location (closest or furthest well) for more than three consecutive trials. There were 10 trials within a session and individuals received 5 sessions. Our measure of performance was the number of correct trials within a session.

### Spatial discrimination

To assess spatial discrimination performances, we used the same two-well apparatus as that in the visual discrimination task (above), but instead of encircling each well with a particular color, both wells were unmarked and identical, differing only in their location on the apparatus (e.g., Pravosudov et al. 2005; Sanford and Clayton 2008; Sewall et al. 2013; Shaw et al. 2015; Shaw et al. 2019). The correct well was furthest from the bird and the incorrect well was closest to the bird. There were 10 trials within a session and individuals received 3 sessions. Our measure of performance was the number of correct trials within a session.

### Spatial ability

Individuals were required to locate a single rewarded well among 10 (2 × 5 grid) unmarked wells. The reward location remained consistent for each individual but the location was counterbalanced across individuals within years; for half of the individuals, the reward was located on the second row (furthest from the bird), second well from the bird’s left, and for the other half of the birds, it was located in the second row, second well from the right. Birds received four training sessions prior to testing in which all 10 wells were uncovered and the location of the rewarded well was visible to ensure that they had experienced the rewarded location. During a test session, all wells were covered with crepe paper and the number of incorrect choices made before locating the rewarded well was recorded. We considered new incorrect choices only and ignored repeated incorrect choices because repeats were not recorded in all years, and thus, learning measures were comparable across years. Therefore, there were a total of nine incorrect choices per trial. There were two trials within a session and individuals received eight sessions. Our measure of performance was the number of incorrect choices within a session.

### Adult breeding

In the March of each year, we recaptured surviving pheasants that had been released in the previous year using baited funnel traps that were checked three times per day. Caught pheasants were housed in outdoor pens which contained multiple shelters, food hoppers, water, and branches for perching. In years 1 and 2, individuals from our released population (for which we had genetic information) were housed in single-male multiple-female groups of either two, three, or four females and in year 3, we had larger groupings of ~15 individuals with approximately 4:1 ratio of females:males. The social composition of pens was held constant while eggs were collected daily until the end of April. Collected eggs were artificially incubated as a single batch in a Brinsea OvaEasy 580 incubator. After 25 days of incubation, hatched chicks were randomly allocated to one of four identical rearing houses (described above). Hence, we were unaware of which chicks had hatched from which egg and therefore from which adult housing pen they came.

### Pedigree

In each year, blood samples were collected when the birds were approximately 10 weeks old, the day before their release into the wild. In year 4, we also collected blood samples from adults held temporarily in captivity that we had not previously reared because these individuals formed the majority of our breeding adults. Across years, we had genetic information for 50% of mothers and 61% of fathers that we housed in captivity during the study (see Supplementary Information 2—*Pedigree Information*). DNA was extracted from blood samples and using data from 15 microsatellite markers (see Supplementary Information S2—*Pedigree Information*, *Genetic analyses* for details), we used Colony software (Jones and Wang 2010) to assign parentage to individuals (see Supplementary Information 2—*Pedigree Information, Colony parameters*). Candidate parents were those individuals that we captured and housed in captivity from whom we collected eggs and had taken blood samples from as chicks (years 1–4) or as adults (year 4).

### Statistical analyses

#### Heritability and correlations between cognitive performances

All analyses were conducted in R version 3.6.1 (R Core Team 2017). An animal modeling approach was used to estimate the genetic parameters of cognitive traits (see Wilson et al. 2010), implemented using the *asreml* package (Gilmour et al. 2009). Detailed modeling methods are fully described in the supplemental materials (see Supplementary Information 3*—Animal models*); so, we keep the present description brief. First, a series of univariate mixed models (including animal models) were compared (using AIC and Likelihood ratio tests) to test for the presence of additive genetic variance in each cognitive trait. Then, estimates of genetic variance were extracted from animal models and scaled by the phenotypic variance (V_P_) to yield estimated heritabilities (proportion of variance explained by additive genetic component V_A_), which we present with associated estimated SE. For univariate models of *visual discrimination, spatial discrimination,* and *spatial ability* (i.e., traits with repeat measures), we employed a random regression strategy for modeling additive genetic and permanent environment effects across repeated sessions (following approaches described in, e.g., Wilson et al. 2005). We used first order (linear) random regressions on session, treated as a continuous variable but rescaled to a maximum of zero (final session). This allowed us to interpret random intercept variances as pertaining to cognitive performance in the final session (see Supplementary Information 3*—Animal models* for further explanation). For *inhibitory control*, there was only a single measure of performance; so, this was used following a square root transformation to better approach the assumption of Gaussian errors. The assumption of Gaussian errors was used in all models and appeared reasonable based on visual inspection of residuals. Fixed effects of sex, rearing group (house/year combination), mean test order, and (where appropriate) session number were included as fixed effects. For each trait, we also calculated the coefficient of variation (the square-root of the V_A_ component divided by the (observed) phenotypic mean; CV_A_ = √V_A_/µ), as an alternative standardization of genetic variance (Houle 1992; Hansen et al. 2011). This is provided for completeness but we suggest it may not be appropriate for the purposes of cross-study comparisons given scale considerations arising from trait definitions (see Discussion). For *visual discrimination, spatial discrimination,* and *spatial ability*, we calculate CV_A_ using the observed mean performance in the final session. For *inhibitory control*, we estimated CV_A_ using additive variance and mean determined from the square root transformed data, but also generated the corresponding estimate using the observed data scale. After fitting univariate models, we sought to estimate the among-individual correlation structure between traits (**ID**) and then to characterize its genetic component (**G**) (see Supplementary Information 3*—Animal models* for full details). Treating *spatial discrimination* as a trait with repeated measures (as per univariate models), we were unable to obtain stable convergence of multivariate models from this data set. Consequently, to reduce the number of parameters, we elected to use the mean observed phenotype (across three sessions) for each individual as the measure of performance. **ID** was then estimated in a four-trait multivariate mixed model with; fixed effects on each trait as described for univariate models; a random effect (intercept) of individual identity on each trait; random slopes on session for *visual discrimination* and *spatial ability* (with session scaled as described above, such that random intercept (co)variances pertain to performance at final observation); and observation level (i.e., residual, interpretable as within-individual) variances fixed to zero for those traits with a single observation (i.e., *inhibitory control, mean spatial discrimination*). The latter is imposed since among- and within-individual variance cannot be partitioned from a single observation per individual. Similarly, as *visual discrimination* and *spatial ability* were not recorded at the same observations, observation level (residual) covariance between these traits is undefined in the data structure and so was not modeled. Pairwise phenotypic correlation estimates (*r*_P_) were obtained from this model and the among-trait correlation structure explored using eigen decomposition. We then used multivariate animal models to partition **ID** into genetic **G** and nongenetic components to estimate the corresponding set of between trait genetic correlations (*r*_G_) and subject these to eigen decomposition. In the current context the eigen decompositions of among-individual phenotypic and genetic correlation structures are equivalent to principle component analyses (PCA) and allow us to determine if correlation structure is consistent with a single underlying latent variable, analogous to a general intelligence model of cognition. Note that all response variables were scaled so that positive values represent good performance. For example, we reversed the performance scores for the inhibitory control and spatial ability tasks by subtracting the number of errors made from the maximum number of errors. This means that under a general intelligence model, correlations among cognitive traits are predicted to be uniformly positive in **ID** and/or **G.**

### Ethics statement

Birds were habituated to human observation and were subject to minimal handling. All procedures were adopted to mitigate stress during cognitive testing and birds could choose whether or not to participate in tasks. Birds were reared at a lower density than that recommended by DEFRA’s code of practice (DEFRA, 2009). During capture of adults from the wild, traps were checked at least three times a day. Adult birds were held in captivity for 3 months, after which they were released back into the wild. All work was approved by the University of Exeter Psychology Ethics Committee and the work was conducted under Home Office licence number PPL 30/3204 to J.R.M.

## RESULTS

Likelihood ratio tests (LRT) and comparison of AIC scores across univariate model formulation support the presence of additive genetic variance in all four traits (Supplementary Information 4—*AIC and LRT univariate model comparisons*). Thus, the preferred model (lowest AIC score) included additive genetic effects for all traits. For *inhibitory control* that was observed only once, the animal model was a significantly better fit than the null model (LRT model 1 vs. model 0; χ ^2^_0,1_ = 2.86, *P* = 0.045). For all other traits, there was evidence of among-individual variance in average performance (LRT of model 1 vs. model 0; all *P* < 0.05) and in variation in rate of performance change over repeat sessions (LRT of Model 1 vs. Model 0; all *P* < 0.05). For *visual discrimination,* stepwise addition of the random genetic intercept (Model 3) and slope (Model 4) led to significantly improved model fits, providing evidence of significant genetic variance that is itself a function of session. For *spatial ability,* a similar conclusion is statistically supported given that Model 4 (random regression animal model) is a significantly better fit than Model 3 (in which the genetic effect is assumed constant across session; Supplementary Information 4—*AIC and LRT univariate model comparisons*). We note that while Model 3 is not actually significantly better than Model 2, Model 4 is (comparison not shown in Supplementary Information table, LRT model 4 vs. model 2; χ ^2^_3_ = 14.93, *P* = 0.002). Thus, the influence of individual genetic merit here only becomes apparent when it is allowed to vary across sessions. Finally, for *spatial discrimination*, stepwise additions result in significant improvement from Models 0 to 3, providing evidence of among-individual variance in random intercept (Model 1 vs. Model 0), slope of regression on session (Model 2 vs, Model 1), and additive genetic variance (Model 3 vs. Model 2). We encountered problems reaching the *asreml* default convergence criteria for Model 4. Nonetheless, given apparent stability of model log-likelihood and parameter estimates after several thousand iterations, we chose to accept the solution as valid. Based on this, there is no statistical support for dependence of additive genetic merit on session (LRT Model 4 vs. Model 3; χ ^2^_2_ = 0.074, *P* = 0.964). Despite the lack of significant genetic slope variance in *spatial discrimination*, we decided for consistency to estimate heritability and repeatability under a “final” model of Model 4 for all traits with repeat measures. Given scaling of the session variable (see earlier), we calculated these using random intercept variances only such that estimates pertain to the final observed session in each case. Heritability of *inhibitory control* was estimated under Model 1.

**Figure 1:**
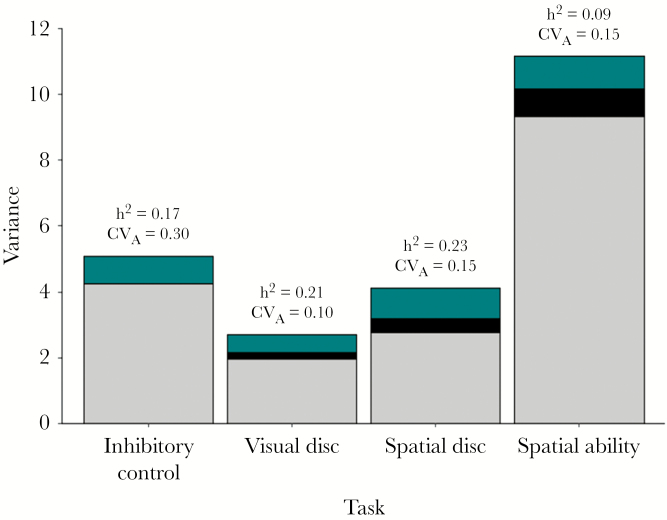
Unstandardized variance components (stacked bars), heritability (h2), and coefficient of variation (CVA) for four cognitive task performances in pheasants. Variance components are additive genetic (VA, green bars), permanent environment effects (VPE, black bars; traits with repeated measures only) and residual variances (VR, gray bars). Variance components for inhibitory control were obtained from a single measure univariate animal model. Inhibitory control was square root transformed. We fitted random regression animal models for visual discrimination, spatial discrimination and spatial ability tasks and show the variance at intercepts, which represents performance by the end of testing taking into account performance in all sessions.

Repeatabilities (with SE) for *visual discrimination*, *spatial discrimination,* and *spatial ability* were 0.28 (0.04), 0.33 (0.08), and 0.17 (0.03), respectively (where R is conditional on fixed effects and estimated as R = V_I_/V_P_ = (V_A_+V_PE_)/V_P_). Across traits, genetics explained between 9% and 23% of the variation. There were moderate heritabilities (at final session) of both discrimination tasks (visual, 21% and spatial, 23%), and lower estimates for *inhibitory control* (17%) and *spatial ability* (9%) (Figure 1). The coefficient of variation was lowest for *visual discrimination* and highest for *inhibitory control* performance (range, 0.10–0.30) (Figure 1). Note that, given convergence issues with Model 4 for *spatial discrimination* we also checked parameter estimates under Model 3 (which was preferred under AIC) and found they were very similar such that the choice of final model here is of little consequence (under Model 3, R = 0.32 (0.05), h^2^ = 0.22 (0.11), CV_A_ = 0.13). We also checked how the square root transformation of *inhibitory control* influenced final estimates (relative to modeling untransformed data) by refitting Model 1 on the observed data scale. This yielded estimates of h^2^ = 0.12 (0.10) and CV_A_ = 0.39. The significance and magnitude of fixed effects varied across task performances. These effects are not directly relevant to hypotheses being tested but are reported in full in the supplemental materials (see Supplementary Information 5—*Estimated fixed effects from final models of Cognitive performance traits, a–d),* as are the estimated variance components under final models of each trait (see Supplementary Table 5e).

### Correlations among cognitive traits

The four-trait mixed model provided evidence of some significant among-individual correlation structure among cognitive performance traits (χ ^2^_13_ = 23.711, *P* = 0.034; Table 2). However, estimated correlations between trait pairs (conditional on fixed effects) were weak, had large standard error, and were not all positive as predicted under a general intelligence model. Eigen decomposition of the correlation matrix reflects this, with the first principle component explaining just 35% of the variation and loading antagonistically on visual discrimination relative to the other traits (see Supplementary Information 6*—PCA*, Supplementary Table SI 6a, 6b).

**Table 2:** Estimated phenotypic (among-individual) and additive genetic correlations among four cognitive traits measured in pheasants

Trait	Inhibitory	Visual disc	Spatial disc	Spatial ability
Inhibitory	*-*	−0.168 (0.120)	0.089 (0.089)	0.147 (0.130)
Visual disc	−0.195 (0.389)	*-*	0.012 (0.137)	−0.162 (0.154)
Spatial disc	0.690 (0.527)	0.657 (0.428)	-	0.244 (0.120)
Spatial ability	0.999 (NA)^a^	0.092 (0.336)	0.999 (NA)^a^	*-*

Phenotypic correlations (above the diagonal) are estimated from a four-trait multivariate mixed model with individual as a random effect. Genetic correlations (below the diagonal) were estimated from one trivariate animal model (dark gray) and three bivariate models (light gray). Standard errors are shown in parentheses.

^a^The model converges at a boundary condition with *r*_G_ constrained to +1 to keep it in allowable parameter space. In this circumstance no SE is estimable.

While we were unable to estimate **G** among all four traits simultaneously (see Supplementary Information 3*—Animal models*), we did manage to estimate the genetic-variance correlation matrix among *inhibitory control, visual discrimination*, and *spatial ability* in a trivariate formulation of the random regression animal model. Based on comparison to a reduced model in which all cross-trait genetic correlation terms were set to zero, there is no evidence for significant genetic correlation among these traits (χ ^2^_8_ = 7.489, *P* = 0.485). Similarly, bivariate models of *spatial discrimination* and each of the other traits provided no evidence of significant genetic correlation structure (*inhibitory control*: LRT = 2.274, *P* = 0.131; *visual discrimination*: LRT = 0.237, *p* = 0.237; *spatial ability*: LRT = 4.411, *P* = 0.110). The lack of significant genetic correlations was despite point estimates of the genetic correlations that were strongly positive in some (but not all) cases (Table 2). Eigen decomposition of the genetic correlation matrix (formed by combining estimates from the trivariate and three bivariate models) suggests 69% of the variance is explained by the first vector, which loads on all traits in the same direction (though less strongly on *visual discrimination* than the other traits; see Supplementary Information 6*—PCA*, Supplementary Table 6c, 6d). Thus, our best estimate of **G** is actually broadly consistent with expectations under the general intelligence model but is characterized by high levels of statistical uncertainty precluding any statistically robust inferences. Note estimates of additive genetic variances for each trait from multivariate models were very similar to those from univariate models (data not shown).

## DISCUSSION

We found evidence of additive genetic variation underpinning all four cognitive traits in pheasants, although estimated heritabilities were low to moderate in all cases. Discrimination of visual and spatial cues had the largest genetic components, compared with inhibitory control and (especially) spatial ability. Investigations of (additive) genetic variance for specific cognitive traits are rare and limited to a few taxa (Croston et al. 2015). As noted earlier, direct comparison of genetic variation levels across traits and studies is not always easy. We focus our discussion on heritabilities (estimated conditional on fixed effects) when trying to place these results in a wider context. It is important to note that heritabilities provide an imperfect tool for comparison and can sometimes give a misleading view of evolutionary potential (see e.g., Wilson 2008; Hansen et al. 2011 for in depth discussion of issues that can arise). Consequently, mean-scaled measures (e.g., CV_A_) are being increasingly advocated for behavioral studies. However, these are only comparable across studies if traits are measured on ratio scale with an objective zero point (Houle 1992; Dochtermann and Royauté 2019). This is not strictly the case here, as cognitive performance can be equally characterized as average success rate or as average failure rate in any task (i.e., the choice of zero, and hence value of the mean phenotype, is an arbitrary decision for the experimenter). While presented estimates of CV_A_ would thus be valid for predicting selection responses of these traits as defined in this population, they are not appropriate metrics for cross-study comparison. We also found some, albeit limited, significant (among-individual) phenotypic correlation structure among traits. Principle component analysis however was not consistent with a strong leading general intelligence factor. Nor was there evidence of significant genetic correlations among traits, although this may be partly due to low statistical power. Towards the end of our Discussion, we make some cautious interpretation of qualitative patterns in both observed phenotypic (among-individual) and genetic covariance structures. We consider what these patterns may mean for responses to selection on cognitive traits and argue that comparing both the phenotypic and genetic correlations is important for our understanding of factors that maintain individual variation in cognitive traits.

Our heritability estimate for the discrimination of binary visual cues (0.21) is similar to estimates obtained for visual learning in insects (Brandes 1988; Lofdahl et al. 1992) but higher than that reported in another galliform, the red junglefowl (Sorato et al. 2018). Fast and accurate learning of discriminations between stimuli (e.g., potential food types or potential predators/competitors) is likely to have important fitness consequences for pheasants and in this context the moderate heritability suggests relatively rapid evolution of discrimination ability could be possible (at least in the absence of constraint arising from genetically correlated traits; Walsh and Blows 2009). The conceptually similar discrimination ability based on spatial position exhibited a marginally higher heritability estimate (0.23). Conversely, we found low heritability of spatial ability (0.09), as measured using a foraging grid. Three recent studies have reported indicators of strong directional selection favoring accurate learning of spatial locations in similar tasks using variants of a foraging grid, with accurate learners surviving for longer (Sonnenberg et al. 2019) and producing more offspring (Branch et al. 2019; Shaw et al. 2019). These studies involved species that are dependent on caching food (chickadees [*Poecile gambeli*] and North Island robins, [*Petroica longipes*]) and thus, these species are expected to have been strongly selected for better spatial memory over generations. Although pheasants do not cache food items, they likely have a strong spatial dimension to their lives including movement between territories and feeders, return to resource-rich areas, and memory of refuges. Speculatively, if strong directional selection has acted on spatial ability in pheasants, this might have eroded standing genetic variation contributing to the low heritability estimate (Falconer and Mackay 1996; Kruuk et al. 2000).

The heritability estimate for inhibitory control was low to moderate (0.17) with relatively high uncertainty (being based on a single observation per individual) though still marginally significant based on likelihood ratio tests. Estimates obtained from animal models are often more conservative than other methods in which common environment effects are difficult to control for (e.g., parent–offspring regression, Kruuk and Hadfield 2007; Wheelwright et al. 2014). This methodological consideration may partially explain why our findings differ so much from the high levels of genetic contribution to inhibitory control variation reported in humans (Friedman et al. 2008; Schachar et al. 2011). Alternatively, our low heritability estimate for inhibitory control may be due to high residual variance associated with age effects. In general, trait heritabilities often vary with age (Wheelwright et al. 2014). We only measured the pheasant’s cognitive performance at a single point, early in life. Prior to testing, birds were raised in a standardized environment (as far as possible). This was important because we have previously shown the development of inhibitory control in pheasants depends on experience (van Horik et al. 2018) and both short (Griffin et al. 2020) and longer-term changes (van Horik et al. 2019) in predictability of the rearing environment. We do not discount the possibility that, for instance, the heritability of inhibitory control would be higher if assayed later in life, but equally, this measure could be confounded by differential experiences for individuals during the intervening time.

Selection does not act on traits in isolation and so relationships between traits will also have consequences for how cognitive variation is maintained and thus, how abilities evolve. Here, we did find some weak phenotypic structure, but this was not underpinned by significant genetic correlation structure. This suggests that the phenotypic correlation may well be due to shared environmental effects acting on the traits rather than underlying genetic factors arising from pleiotropy or linkage disequilibrium. However, we stress that these results are to be interpreted with caution because the large standard errors on estimated genetic correlations and the inability to estimate the error in some cases, suggests limitation of our statistical power. In other words, we cannot statistically reject Cherverud’s conjecture that **G** matches the (among-individual) phenotypic correlation structure (Cheverud 1988). Quantitative genetic studies require large volumes of data to achieve high precision for genetic correlation estimates (Wilson et al. 2010), and this becomes increasingly difficult when genetic variance for traits is low. Here, larger sample sizes would clearly have helped, although this high-throughput phenotyping poses a major challenge when measuring cognitive performance traits. Below, we discuss the qualitative patterns emerging from our estimates of phenotypic and genetic correlation structure, while reiterating the caveat that there was no statistical support for significant genetic correlations.

Principle components analysis of the estimated (among-individual) phenotypic correlation matrix revealed no single dominant leading vector, with each of the four axes explaining between 18 and 35% of the variation. This is inconsistent with a general intelligence *(g)* model of cognition as applied to variation at the among-individual level. That is because under such a model, we would expect all traits to load strongly (and in the same direction) onto a dominant first principle component (Plomin 2001; Plomin and Spinath 2002). The more modular structure of cognition indicated by our results supports our previous findings (derived from phenotypic correlations within 1 year), that the emergence of a single factor that explained the majority of the variance was highly susceptible to test battery composition based on six of a potential nine tasks (van Horik, Langley, Whiteside, Laker, et al. 2018) providing little support for *g* in pheasants. In this present study, all traits except performance in the visual discrimination task, loaded with the same sign onto PC1. This provides a qualitative indication that the ability to discriminate between visual cues may be distinct from learning about locations or inhibiting behavior. This is similar to song sparrows (*Melospiza melodia*) in which visual learning ability did not positively correlate with inhibitory control (Boogert et al. 2011). Conversely, in New Zealand robins (Shaw et al. 2015) and Australian magpies (*Cracticus tibicen dorsalis*) (Ashton et al. 2018), a general intelligence model of cognition was supported.

In contrast to the phenotypic correlation structure where we found weak relationships between task performances, the first principle component of the genetic correlation matrix explained 69% of the variation, with all four traits having same-sign loadings. Taking the point estimates at face value means that our best estimate of **G** is actually consistent with the general intelligence model of cognition (Plomin and Spinath 2002; Burkart et al. 2016). In fact, four of the six possible pairings between tasks exhibited a strong positive genetic correlation (*r*_G_ > 0.66), albeit not a statistically significant one. For instance, the discrimination tasks exhibited positive genetic correlation (*r*_*G*_ = 0.66), which is not surprising given both tasks required individuals to discriminate between two cues. It is therefore intuitive that both tasks would involve similar cognitive processes, such as comparable working memory capacity and levels of attention. However, despite being genetically correlated, they were not phenotypically correlated (*r* < 0.01). Similar considerations apply to the spatial tasks, which we had expected to covary both phenotypically and genetically because both tasks assessed an individuals’ ability to learn about and respond differently to different locations. The phenotypic correlations between these traits was low (*r* = 0.23), while the genetic correlation estimate was almost 1 (but highly uncertain). More generally the apparently poor correspondence between phenotypic and genetic correlation estimates may simply arise because of high uncertainty in the latter. However, to the extent that apparent differences are real, they also suggest that environmental factors may differentially affect how individual cognitive abilities are expressed, thus masking a genetic basis of variation that is common to the different cognitive traits. We do know, for instance, that recent negative social experiences are related to poorer performances on the spatial discrimination tasks in adult pheasants (Langley, et al. 2018), but whether similar effects on discriminating between color cues also occur has yet to be investigated.

Understanding how selection may act on cognitive traits is not without difficulty. We found that a suite of cognitive performance traits exhibited by pheasants, which had been exposed to selection on survival, varied among individuals in part due to heritable variation. Heritabilities were low to moderate across the four traits and, while some phenotypic correlation structure was apparent, there was no statistical support for genetic correlations. Nonetheless, an apparent disparity between estimated phenotypic and genetic correlation patterns leads us to cautiously suggest that environmental factors may impact different cognitive abilities in differing ways. If so, studies investigating correlation structures among cognitive traits should be cautious if seeking to make evolutionary (genetic) inferences from phenotypic patterns. As a final note, although psychometric tasks aim to test a single, discrete cognitive ability, performance in such tasks is likely the result of various interacting cognitive processes (e.g., attention, working memory, long-term memory), each of which may be influenced by the expression of multiple genetic loci. This makes it difficult to isolate which trait or suite of traits are actually heritable because genetic variance detected in task performances could be due to any or all of these factors (see Smulders 2015). Additionally, cognitive performance is not only a consequence of cognitive ability but is also affected by motivation (Rowe and Healy 2014), neophobia (Guido et al. 2017), and stress responsiveness (de Kloet et al. 1999; Mendl 1999), among other factors. The interaction of potentially numerous genetic and nongenetic mechanisms may maintain variation in cognitive abilities even when traits are under strong directional selection.

## Supplementary Material

araa029_suppl_Supplementary_Information_1

araa029_suppl_Supplementary_Information_2

## Notes

The authors are grateful to Seb Bekker, James Foley, Rachel Peden, Sara Raj Pant, Jenny Coomes, Kenzie Bess, Lucy Capstick, Molly Watts, Heather Warrender, Elena Zwirner, Joe Wilde, and Camille Troisi for their assistance with bird husbandry and/or the collection of cognitive data. Thank you to Rothamsted Research for access to their land.

## Notes

Data accessibility: Analyses reported in this article can be reproduced using the data provided by Langley et al. (2020).
