# Supplementary material for: Heritability and correlations among learning and inhibitory control traits
Source: Behav Ecol. 2020 Mar 29;31(3):798–806. doi: 10.1093/beheco/araa029 (PMC7428062; doi:10.1093/beheco/araa029)
Supplement: araa029_suppl_Supplementary_Information_1 [file araa029_suppl_supplementary_information_1.docx]

**Supplementary Information – Heritability and correlations among learning and inhibitory control traits**

**SI 1 – Cognitive tasks**

**SI 1a. Inhibitory control task**

**SI 1b. Learning tasks**

**SI 2 – Pedigree information**

**SI 3 – Animal models**

**SI 4 – AIC and LRT univariate model comparisons**

**SI 5 – Estimated fixed effects from final models of cognitive performance traits**

**SI 5a. Inhibitory control**

**SI 5b. Visual discrimination**

**SI 5c. Spatial discrimination**

**SI 5d. Spatial ability**

**SI 6 – Principle components analyses (eigen decomposition) of phenotypic (among-individual) and additive genetic correlation matrices**

**SI 1 – Cognitive tasks**

**SI 1a - Inhibitory control task**

The detour reach task was a plastic cylinder, 8 cm in length (Years 1 and 2: open at both ends; Year 4: open at one end) fixed to a plastic base, within which was a centrally placed food reward. Birds received four training sessions in which they were presented with an opaque version of the task (wrapped in black tape) requiring them to learn the motor action of reaching behind the barrier to acquire food. Individuals were then presented with a test trial in which food was placed within a transparent version of the apparatus. Only individuals to complete a minimum of 3 of 4 training sessions and retrieve the food in the test session were included in analyses (Table SI 1a). This ensured that individuals were comparable in their experience of reaching within the tube before testing and were equally motivated to retrieve the food reward during the test.

**Table SI 1a: Sample sizes for participating individuals of the inhibitory control task (detour reach)**

| **Year** | **N individuals** | **n completed training** | **n completed test** | **n completed training and testing** |
| --- | --- | --- | --- | --- |
| 1 | 199 | 135 | 156 | 125 |
| 2 | 195 | 187 | 161 | 157 |
| 4* | 95 | 66 | 92 | 59 |
|  |  |  | **Total =** | **341** |

* Only data from birds that were not exposed to transparent objects prior to the inhibitory control test session (see van Horik *et al.* 2018) were used for analyses, thus maintaining consistency in testing conditions across years.

**SI 1b - Learning tasks**

**i. ii. iii.**

**Figure SI 1b: Aerial view of pheasant chick relative to the foraging grid apparatus used to test i) visual discrimination; ii) spatial discrimination; and iii) spatial ability. The black ‘+’ was not visible on the apparatus but represents the rewarded well (correct) during a trial and the unmarked wells were unrewarded and blocked with card so they could not be pecked through (incorrect).**

**SI 2 – Pedigree information**

In year 1, pheasant chicks were purchased from a commercial game dealer. Hence, we do not have parentage information on these individuals. These chicks were released into the wild at 10 weeks old and surviving individuals were caught and held temporarily in captivity for breeding in the following year and thus, are classified as only ‘parents’. In years 2 and 3, captured adults males were housed singly with 2, 3 or 4 females and in year 4 we had four larger groups of 15, consisting of 11 females and 3 males. Across years, 33% of the captured adult males and 11% of adult females were those that we reared as chicks and had collected blood samples from. These individuals were therefore included in analyses as both parents and offspring but only classified as one of these categories within a single year. In year 4, we also collected blood samples from adults that we did not rear as chicks and therefore had total genetic information for 89% of mothers and 56% of fathers. Across years, we had genetic information for 50% of mothers and 61% of fathers that we housed in captivity during the study.

Genetic analyses

Approximately 50 µl of blood was taken from the brachial vein of each individual and added to an eppendorf containing 1.5ml of analytical reagent grade absolute ethanol. Blood was stored in screw-topped rubber-sealed eppendorfs in a refrigerator at 2 °C. DNA was extracted from the blood samples using an ammonium acetate precipitation method (Nicholls *et al.* 2000; Richardson *et al.* 2001). Individuals were genotyped at 15 autosomal *Phasianus colchicus* microsatellite loci in three plexes: Plex 1 (Ph12883, Ph8073, Ph18331 and Phconsgr542), Plex 2 (Ph18799, Phconsgr157, Ph6001, Phconsgr99 and Ph22803), plex 3 (Ph10573, Phconsgr540, Ph9841, Ph15450, Ph5048 and Ph7712) (see Table SI 2a. Microsatellite marker information). The combined non-exclusion probability (first parent) of the set was *p*=0.0018 (based on 24 unrelated individuals at 15 loci and calculated using Cervus v 3.0.7; (Kalinowski *et al.* 2007). PCR amplification was performed using the Qiagen Multiplex PCR Plus kit (Applied Biosystems) with the forward primer 5’ end-labelled with a fluorescent dye (HEX, 6-FAM or NED) and following the manufacturer’s protocol, but with the DNA lyophilized and a 2-µl reaction volume used, following (Kenta *et al.* 2008). A drop of mineral oil was placed over each PCR reaction prior to PCR. PCR amplifications were performed using a DNA Engine Tetrad 2 Peltier Thermal Cycler (Bio-Rad, Hercules, USA) in three multiplex sets with no overlapping allele lengths of the same dye colour (see Table SI 2a. Microsatellite marker information). The following hot start PCR profile was used: 95 °C for 15 min, followed by 44 cycles of 94 °C for 30 s, 57 °C for 90 s, 72 °C for 60 s and a final step of 60 °C for 30 min. PCR products were separated on an ABI 3730 DNA Analyser with an ABI ROX500 size standard and alleles scored using GENEMAPPER 5.0 (Applied Biosystems) DNA fragment analysis software.

Pheasants are easily sexed from 1-day-old using wattle presence as a cue (Woehler & Gates 1970) and are sexually dimorphic in body size and plumage by ~4-weeks-old (Whiteside *et al.* 2017). To confirm these observations we used two genetic sex-typing markers to identify the sex of the birds and to check for any mistakes in any sexes assigned based on phenotype (Z002B: (Dawson 2007) and P2-P8: (Griffiths *et al.* 1998)). Each sex-typing markers required its own unique marker-specific PCR program for amplification and for this reason Z002B and P2-P8 were amplified separately and separate to the microsatellites loci that had been amplified in multiplexes. The PCR programs used with the sex markers were as follows, Z002B: 95 °C for 15 min, followed by 34 cycles of 94 °C for 30 s, 56 °C for 90 s, 72 °C for 60 s and a final step of 60 °C for 30 min and for the P2-P8 marker: 95 °C for 15 min, followed by 44 cycles of 94 °C for 30 s, 49 °C for 90 s, 72 °C for 90 s and a final step of 72 °C for 10 min. The products amplified with Z002B were added to the Plex 2 microsatellite amplicons and P2-P8 products were added to the Plex 1 microsatellite amplicons post-PCR for each individual, and this combined pooled of PCR product was loaded on the ABI3730 DNA Analyser. All 15 microsatellite loci were confirmed autosomal based on the observation that they amplified in both males (ZZ) and females (ZW) and no loci were heterozygote deficient in females (a minimum of 660 individuals were genotyped (317 females and 343 males) per locus). A lack of heterozygosity in females would indicated Z-linkage, and failure to amplify in males (ZZ) would suggest W-linkage.

*Colony parameters*

Colony software (<http://www.zsl.org/science/software/colony>) was used to infer parentage (Jones & Wang 2010). In cases in which genotype information is missing, Colony assigns ‘dummy’ parents so that sibship can be inferred. Mating systems were set to ‘polygamy’ for both females and males. It is suggested to only include ‘with in-breeding’ in cases where strong inbreeding is known. Therefore, we selected ‘without inbreeding’ for all years. We also selected ‘without clones’ as pheasants cannot reproduce asexually, and ‘diploid’. We selected the ‘maximum’ length of run and ‘full likelihood’ analysis method to ensure highest accuracy of parentage. The run specifications were set to ‘No’ for the updating of allele frequency; ‘yes’ for sibship scaling, ‘1’ run and ‘1234’ random number seed. ‘No prior’ was selected for sibship prior because we had no knowledge of mean sibship size. We had no known maternal or paternal sibships, and ‘no’ excluded maternity, paternity or sibships.

*Outputs*

Colony returns a list of the most likely maternity and paternity identities (with associated individual-level likelihoods) and the best maximum likelihood configuration (of parent pairs) for each offspring. Colony assigned 175 maternities (likelihood, 0.19 Min – 0.99 Max) and 328 paternities (likelihood, 0.42 Min – 0.99 Max) from provided genotypes with a mean likelihood of 0.99 (Table SI 2.b). There were also 101 dummy maternities and 66 dummy paternities based on sibships.

**Table SI 2.b: Probability ranges for inferred parent pairs in each year**

| **Year** | **Probability range** |
| --- | --- |
| **2** | Mean 0.99, median 1, IQR: 0 |
| **3** | Mean 1, median 1, IQR: 0 |
| **4** | Mean 0.99, median 1, IQR: 0 |

*Final pedigree*

Parents were included in the pedigree file based on the best maximum likelihood configuration output from Colony. We used the Pedantics (Morrissey & Wilson 2010) R package to obtain pedigree statistics. The final pedigree structure contained 985 informative individuals, with 542 known maternities and paternities. There were 669 full sibling pairs and an additional 460 and 2112 half-sibling pairs, respectively. The mean maternal and paternal sibship sizes were 3.4 and 5.6. The pedigree had a depth of 4 generations.

**SI 3 – Animal models**

*Univariate model overview*

For each cognitive ability, we first fitted a series of univariate mixed models differing in random effect structure (described below) to test for and characterise both among-individual and additive genetic variance. Response variables were the number of correct choices within a session for the learning tasks and the number of errors made within a single trial for the inhibitory control task. Inhibitory control performances were square root transformed to normalize residuals. We estimated genetic variance using an animal model approach, in which the inverse of the pedigree-derived additive genetic relatedness matrix to solve the additive genetic variance. For traits observed on individuals across repeat sessions, this included use of random regression animal models (RRAM; see below). We assume Gaussian errors in all cases and, for each trait, compared models of differing random effect structure using AIC. All models were fitted using ASReml_R (Butler 2019) and heritabilities (with SE) were estimated as the ratio of additive genetic variance (V_A_) to phenotypic variance (V_P_) using the ‘vpredict’ function. We estimated V_P_ conditional on fixed effects as the sum of the estimated random effect variances (for random intercepts only under random regression) and residual variance V_R_.

*Fixed effect structure*

For all models of all traits we included sex, year, house, and mean test order (calculated from the first 20 entrances to the testing chamber) as fixed effects to control for phenotypic variance caused by these factors. The inclusion of year controlled for slight differences between years in experimenters and task apparatus (see SI 1 – *Cognitive tasks*). We also included a linear function of ‘session’ for those traits where trials were conducted across multiple repeated sessions per individual. We used Wald F tests to assess the significance of fixed effects but elected not to perform model simplification as we wanted heritability estimates to be conditioned on a common set of fixed effects and thus comparable across abilities.

Note that in year 4, subjects were exposed to one of two different housing conditions: a spatially unpredictable environment (barriers that moved daily) in 2 houses, while the other 2 houses were exposed to a spatially predictable environment (constant barriers), as part of a separate experiment (van Horik *et al.* 2019). Although this treatment can influence inhibitory control ability, preliminary analysis of the current data with separate general linear models (not shown) showed no significant effect of this treatment on performance for the 3 tasks conducted in year 4 (Inhibitory control: GLM: LRT = 1.666, *p* = 0.197; Visual discrimination: LRT = 1.386, *p* = 0.324; Spatial ability: LRT = 0.886, *p* = 0.347). Thus, while our models include fixed effects of house and year, we elected not to also include the predictable/unpredictable housing treatment.

*Random effect structures tested in univariate models*

For those traits with repeated measures (i.e. *visual discrimination*, *spatial discrimination* and *spatial ability*) which had multiple testing sessions per individual), we initially attempted to model among-individual and genetic variation using random regressions. Specifically, we fitted a nested series of five models to each trait: (0) no random effects; (1) a random intercept of individual identity; (2) a random regression of individual identity on session number; (3) a random regression of the permanent environment (PE i.e. individual level non-genetic) effect on session number with a random additive genetic intercept and (4) random regressions on session for both PE and additive genetic effects. By modelling the random individual (and genetic) deviations from fixed effect mean performance as linear functions of *session* the model allows variance components for the observed trait (number of trials in which the individual was correct) to change with *session*. Where included in the random effect structure session was scaled to a maximum of zero (i.e. final session=0). This means that the estimated individual, permanent environment, and/or genetic intercept variances are directly interpretable as the corresponding variance at the final observation. We focus on these estimates (rather than, for instance variance in random regression slopes, or predictions of observed trait variance at earlier sessions). Biologically, this means final session performance is the measure of cognitive ability, and this is predicted using all available data, rather than just using the final observation per individual.

In practice, we were unable to obtain stable convergence for the random regression models of *spatial discrimination*, most likely as a consequence of the smaller number of sessions in which phenotypes were observed (just 3 as compared to 5 and 8 for *visual discrimination*, and *spatial ability* respectively). We therefore elected to simplify the spatial discrimination performance to a single measure per individual using the mean phenotype across trials. Additionally, the final cognitive trait (*inhibitory control*) was observed only once per individual. Thus, for these two aspects of cognition we present analyses of response variables with no repeat measures. And simply compared a model (0) with no random effect to (1) a model with a random additive genetic intercept.

*Multivariate models*

We then formulated multivariate models to estimate the genetic correlation structure among traits. We did this using general correlation matrices in ASReml-R, meaning relationships among traits were estimates directly on a correlation scale (as opposed to being estimated as covariances and then rescaled). Response variables and fixed effects were as described above. We first fitted the four trait model including a random effects of individual identity to estimate the (among-individual) phenotypic correlation structure (which we denote as **ID**). Note that since each cognitive ability was assayed separately, observation-level residual (‘within-individual’) correlation terms for traits with repeated observations are not identifiable and were set to zero. Based on univariate model comparisons, random regressions on session were included for *visual discrimination* and *spatial ability* but we focus here only on the correlations of the random intercepts (i.e. individual performance at final session) of these with other traits. Residual (observation level, within-individual) variances were included for these two repeat measures traits, but no residual covariance (since the traits were not recorded at the same observations so this term is undefined in the data structure). For more information and a didactic treatment of how to set up multivariate mixed models in this scenario when some traits have repeat measures and others do not, see Houslay & Wilson (2017) and associated tutorial materials.

Having fit this model, we performed a global test for among-individual correlation by refitting a constrained version of the model assuming all cross-trait correlations in **ID** were zero. This was then compared to the unconstrained model by likelihood ratio test. We consider individual elements of correlation matrix **ID** (i.e. pairwise trait correlation estimates) to be nominally significant if |r| > 1.96*SE. We also subjected this correlation matrix estimate to eigen vector decomposition (PCA) to qualitatively assess congruence with predictions from a general intelligence factor model. Specifically, if among-individual variance in cognitive abilities is underpinned by a single factor we would expect (i) uniformly positive correlation structure in **ID** (given trait definitions) and (ii) the correlation matrix to be dominated by a first eigen vector loading on all traits with the same sign.

Finally, we added additive genetic effects to the multivariate model to partition among-individual (co)variance **ID**into additive genetic (**G**) and non-genetic components. Note that since only some traits have repeat measures, the non-genetic component of **ID** is strictly interpretable as neither the total environmental covariance nor the ‘permanent environment’ covariance structure. However, we do not seek to interpret it further regardless.

**G** was estimated on the correlation scale and first order random regressions on scaled session were included for additive genetic and permanent environment effects of *visual discrimination* and *spatial ability*. Again, we focus only on terms relating to the random intercepts of these two traits which represent (genetic) merit for final performance. The intent was for **G**to be then tested for significant correlation structure and subjected to eigen decomposition exactly as above. However, in practice we were unable to obtain stable convergence in the four trait model. We therefore estimated a ‘composite’ comprised of the estimate from a three trait model (*visual discrimination*, and *spatial ability, inhibitory control;*with random regressions for permanent environment and additive genetic effects for the first two traits), and then estimated genetic correlations (r_G_) with *spatial discrimination* using bivariate animal models. The resulting composite estimate of **G**was nonpositive definite, so we ‘bent’ it to the nearest positive definite correlation matrix using *nearPD* in the package Matrix (Bates & Maechler 2019), before performing the eigen decomposition of the corresponding correlation structure.

**SI 4 –LRT and AIC univariate model comparison**

**Table SI 4a: AIC values and likelihood ratio test comparisons across univariate models for each cognitive trait**

| **Trait** | **Model** | **AIC** | **LnL** | **LRT Comparison** | **Χ^2^** | **DF** | **P** |
| --- | --- | --- | --- | --- | --- | --- | --- |
| *Inhibitory control* | 0 | 915.663 | -456.832 |  |  |  |  |
|  | 1 | 914.803 | -455.401 | 0 vs 1 | 2.861 | 0,1 | 0.045 |
| *Visual discrimination* | 0 | 4548.805 | -2273.402 |  |  |  |  |
|  | 1 | 4461.472 | -2228.736 | 0 vs 1 | 89.332 | 0,1 | 0 |
|  | 2 | 4428.418 | -2210.209 | 1 vs 2 | 37.054 | 2 | 0 |
|  | 3 | 4415.613 | -2202.807 | 2 vs 3 | 14.804 | 0,1 | 0 |
|  | 4 | 4409.998 | -2197.999 | 3 vs 4 | 9.616 | 2 | 0.008 |
| *Spatial discrimination* | 0 | 1985.032 | -991.516 |  |  |  |  |
|  | 1 | 1885.742 | -940.871 | 0 vs 1 | 101.290 | 0,1 | 0 |
|  | 2 | 1878.439 | -935.219 | 1 vs 2 | 11.303 | 2 | 0.004 |
|  | 3 | 1874.487 | -932.243 | 2 vs 3 | 5.952 | 0,1 | 0.007 |
|  | 4 | 1878.413 | -932.206 | 3 vs 4 | 0.074 | 2 | 0.964 |
| *Spatial ability* | 0 | 12243.348 | -6120.67 |  |  |  |  |
|  | 1 | 12171.579 | -6083.790 | 0 vs 1 | 73.768 | 0,1 | 0 |
|  | 2 | 12151.832 | -6071.916 | 1 vs 2 | 23.748 | 2 | 0 |
|  | 3 | 12153.831 | -6071.916 | 2 vs 3 | 0 | 0,1 | 0.5 |
|  | 4 | 12142.897 | -6064.449 | 3 vs 4 | 14.934 | 2 | 0.001 |

**SI 5 – Estimated fixed effects from final models of cognitive performance traits**

1. ***Inhibitory control***

The mean ± se number of errors made in the inhibitory control task was 16.05 ± 0.06. There was a significant effect of sex and rearing group on inhibitory control ability but no significant effect of test order (Table SI 5a).

**Table SI 5a: Fixed effect estimates from Model 1 (animal model) of *Inhibitory control* ability (square root transformed).**

| **Variable** | **Estimate** | **SE** | ***F*** | **DF** | ***p*** |
| --- | --- | --- | --- | --- | --- |
| **Sex**  Male  Female | 0.00  0.55 | 0.26 | **3.56** | **1, 327.2** | **0.034** |
| **Rearing group**  Year 1A  Year 1B  Year 1C  Year 1D  Year 2A  Year 2B  Year 2C  Year 2D  Year 3A  Year 3B  Year 3C  Year 3D  Year 4A  Year 4B  Year 4C  Year 4D | 0.00  -0.52  -0.19  -0.07  -1.56  -2.57  -3.06  -2.01  NA  NA  NA  NA 0.00  -0.09  0.69  0.00 | 0.54  0.52  0.64  0.51  0.53  0.53  0.54  0.61  0.55 | **9.51** | **9, 284.5** | **< 0.001** |
| **Test order** | 0.02 | 0.01 | 3.04 | 1, 327.6 | 0.082 |

1. ***Visual discrimination***

The mean number of correct choices made in the first and final sessions of the visual discrimination task were 4.63 and 7.52, respectively, indicative of learning. There were significant effects of session, and rearing group but no effect of sex or test order on performances (Table SI 5b).

**Table SI 5b:** **Fixed effects from Model 4 (random regression animal model) of *Visual discrimination* performances**

| **Variable** | **Estimate** | **SE** | ***F*** | ***DF*** | ***p*** |
| --- | --- | --- | --- | --- | --- |
| **Session** | 0.67 | 0.03 | **395.8** | **1, 71** | **< 0.001** |
| **Sex**  Male  Female | 0.00  -0.05 | 0.09 | 0.40 | 1, 426.2 | 0.569 |
| **House**  Year 1A  Year 1B  Year 1C  Year 1D  Year 2A  Year 2B  Year 2C  Year 2D  Year 3A  Year 3B  Year 3C  Year 3D  Year 4A  Year 4B  Year 4C  Year 4D | NA  NA  NA  NA  0.00  -0.11  -0.20  -0.24  NA  -1.20  -1.18  NA -0.38  -0.86  -0.97  -0.64 | 0.20  0.20  0.20  0.20  0.20  0.22  0.22  0.22  0.22 | **8.00** | **9, 367.6** | **< 0.001** |
| **Test order** | -0.01 | 0.01 | 2.70 | 1, 445.4 | 0.101 |

1. ***Spatial discrimination***

The mean number of correct choices made in the first and final sessions of the spatial discrimination task were 5.34 and 7.16, respectively, indicative of learning. There was a significant effect of session, rearing group and test order on spatial discrimination performances but no effect of sex (Table SI 5c).

**Table SI 5c:** **Fixed effects from Model 4 (random regression animal model) of *Spatial discrimination* performances**

| **Variable** | **Estimate** | **SE** | ***F*** | ***DF*** | ***p*** |
| --- | --- | --- | --- | --- | --- |
| **Session** | 0.86 | 0.08 | **103.10** | **1, 39.5** | **<0.001** |
| **Sex**  Male  Female | 0.00  0.10 | 0.22 | 0.26 | 1, 293.1 | 0.645 |
| **Rearing group**  Year 1A  Year 1B  Year 1C  Year 1D  Year 2A  Year 2B  Year 2C  Year 2D  Year 3A  Year 3B  Year 3C  Year 3D  Year 4A  Year 4B  Year 4C  Year 4D | NA  NA  NA  NA  0.00  0.65  -0.25  0.71  0.00  NA  NA  0.14 NA  NA  NA  NA | 0.38  0.36  0.37  0.39 | **4.54** | **5, 211.8** | **<0.001** |
| **Test order** | -0.03 | 0.01 | **4.09** | **1, 242.5** | **0.044** |

1. ***Spatial ability***

The mean number of errors made in the first and final sessions of the spatial ability task were 8.42 and 6.87, respectively, indicative of learning. There was a significant effect of year and house on spatial learning performances but no effect of sex or test order (Table SI 5d).

**Table SI 5d:** **Fixed effects from Model 4 (random regression animal model) of *Spatial ability***

| **Variable** | **Estimate** | **SE** | ***F*** | **DF** | ***p*** |
| --- | --- | --- | --- | --- | --- |
| **Session** | 0.20 | 0.03 | **31.50** | **1, 82.6** | **< 0.001** |
| **Sex**  Male  Female | 0.00  0.10 | 0.15 | 0.00 | 1, 441.6 | 0.501 |
| **Rearing group**  Year 1A  Year 1B  Year 1C  Year 1D  Year 2A  Year 2B  Year 2C  Year 2D  Year 3A  Year 3B  Year 3C  Year 3D  Year 4A  Year 4B  Year 4C  Year 4D | NA  NA  NA  NA  0.00  2.43  0.36  1.56  1.01  0.00  0.00  -0.24 1.78  1.28  1.23  1.35 | 0.32  0.31  0.31  0.30  0.31  0.31  0.31  0.32  0.30 | **13.64** | **9, 440.4** | **< 0.001** |
| **Test order** | -0.00 | 0.01 | 0.02 | 1, 442.1 | 0.671 |

**Table SI 5e: Estimated variance, covariance and correlation parameters from final univariate models of each cognitive trait**

| **Trait (Final model)** | **Variance/covariance/correlation** | **Estimate (SE)** |
| --- | --- | --- |
|  |  |  |
| *Inhibitory control (1)* | **V_A_** | 0.84 (0.64) |
|  | **V_R_** | 4.25 (0.67) |
|  |  |  |
| Visual discrimination (4) | **V_A_intercept_** | **0.56 (0.23)** |
|  | V_A___slope_ | 1.17 (0.52) |
|  | r_G­_intercept.slope_ | 0.64 (0.17) |
|  | **V_PE_intercept_** | **0.19 (0.21)** |
|  | V_PE___slope_ | 0.57 (0.51) |
|  | r_PE_intercept.slope_ | 0.50 (0.47) |
|  | **V_R_** | **1.96 (0.08)** |
|  |  |  |
| Spatial discrimination (4) | **V_A_intercept_** | **0.95 (0.61)** |
|  | V_A___slope_ | 0.01 (0.66) |
|  | COV_A­_intercept.slope_ | 0.07 (0.49) |
|  | **V_PE_intercept_** | **0.40 (0.65)** |
|  | V_PE___slope_ | 0.86 (1.04) |
|  | COV_PE_intercept.slope_ | -0.59 (0.63) |
|  | V_R_ | 2.78 (0.25) |
|  |  |  |
| Spatial ability (4) | **V_A_intercept_** | **1.02 (0.55)** |
|  | V_A___slope_ | 3.86 (1.57) |
|  | r_G­_intercept.slope_ | 1.00 (NA) |
|  | **V_PE_intercept_** | **0.82 (0.57)** |
|  | V_PE___slope_ | 0.36 (1.53) |
|  | r_PE_intercept.slope_ | -0.22 (2.07) |
|  | **V_R_** | **9.33 (0.25)** |

Note that under random regression animal models (Model 4), additive genetic (A) and permanent environment (PE) intercept variances can be interpreted as the corresponding component of variance in performance at final session. Total phenotypic variance (conditional on fixed effects) at final session is calculated as the sum of components shown in bold font. Slope variances are in different units and not of directly comparable magnitude. For visual discrimination and spatial ability the relationship between slope and variance at both PE and A levels was modelled on a correlation (r) scale. For Spatial discrimination an equivalent model structure was fitted but with a different parameterisation such that slope-intercept covariances (COV) rather than correlations were estimated.

**SI 6 – Principle components analyses (eigen decomposition) of phenotypic (among-individual) and additive genetic correlation matrices**

**Table SI 6a: Phenotypic variance (%) explained by four principle components of P (on a correlation scale)**

| **1** | **2** | **3** | **4** |
| --- | --- | --- | --- |
| 0.35 | 0.26 | 0.21 | 0.18 |

**Table SI 6b: Loadings of four cognitive traits onto four principle components of P (on a correlation scale)**

|  | **1** | **2** | **3** | **4** |
| --- | --- | --- | --- | --- |
| **Inhibitory control** | -0.489 | 0.335 | -0.800 | 0.091 |
| **Visual disc** | 0.426 | -0.634 | -0.475 | 0.438 |
| **Spatial disc** | - 0.455 | -0.663 | -0.067 | -0.591 |
| **Spatial ability** | -0.611 | -0.216 | 0.360 | 0.672 |

**Table SI 6c: Additive genetic variance (%) explained by principle components of G (on a correlation scale)**

| **1** | **2** | **3** | **4** |
| --- | --- | --- | --- |
| 0.69 | 0.31 | <0.001 | <0.001 |

**Table SI 6d: Loadings of four cognitive traits onto principle components of G (on a correlation scale)**

|  | **1** | **2** | **3** | **4** |
| --- | --- | --- | --- | --- |
| **Inhibitory control** | -0.520 | -0.423 | 0.721 | 0.175 |
| **Visual disc** | -0.180 | 0.840 | 0.430 | -0.276 |
| **Spatial disc** | -0.580 | 0.293 | -0.402 | 0.645 |
| **Spatial ability** | -0.601 | -0.169 | -0.365 | -0.691 |

**References**

Bates, D. & Maechler, M. (2019). Matrix: Sparse and Dense Matrix Classes and Methods. R package version 1.2-17. https://cran.r-project.org/package=Matrix.

Butler, D. (2019). asreml: Fits the Linear Mixed Model. R package version 4.1.0.106. https:// www.vsni.co.uk.

Dawson, D.A. (2007). Genomic analysis of passerine birds using conserved microsatellite loci. University of Sheffield.

Griffiths, R., Double, M.C., Orr, K. & Dawson, R.J.G. (1998). A DNA test to sex most birds. *Mol. Ecol.*, 1071–1075.

van Horik, J.O., Beardsworth, C.E., Laker, P.R., Langley, E.J.G., Whiteside, M.A. & Madden, J.R. (2019). Unpredictable environments enhance inhibitory control in pheasants. *Anim. Cogn.*, 22, 1105–1114.

van Horik, J.O., Langley, E.J.G., Whiteside, M.A., Laker, P.R., Beardsworth, C.E. & Madden, J.R. (2018). Do detour tasks provide accurate assays of inhibitory control? *Proc. R. Soc. B Biol. Sci.*, 285.

Houslay, T.M. & Wilson, A.J. (2017). Avoiding the misuse of BLUP in behavioural ecology. *Behav. Ecol.*, 28, 948–952.

Jones, O.R. & Wang, J. (2010). COLONY: A program for parentage and sibship inference from multilocus genotype data. *Mol. Ecol. Resour.*, 10, 551–555.

Kalinowski, S.T., Taper, M.L. & Marshall, T.C. (2007). Revising how the computer program CERVUS accomodates genotyping error increases success in paternity assignment. *Mol. Ecol.*, 16, 1099–1106.

Kenta, T., Gratten, J., Haigh, N.S., Hinten, G.N., Slate, J., Butlin, R.K., *et al.* (2008). Multiplex SNP-SCALE: a cost-effective medium-throughput single nucleotide polymorphism genotyping method. *Mol. Ecol. Resour.*, 8, 1230–1238.

Morrissey, M.B. & Wilson, A.J. (2010). Pedantics: An r package for pedigree-based genetic simulation and pedigree manipulation, characterization and viewing. *Mol. Ecol. Resour.*, 10, 711–719.

Nicholls, J.A., Double, M.C., Rowell, D.M. & Magrath, R.D. (2000). The evolution of cooperative and pair breeding in thornbills Acanthiza (Pardalotidae). *J. Avian Biol.*, 31, 165–176.

Richardson, D.S., Jury, F.L., Blaakmeer, K., Komdeur, J. & Burke, T. (2001). Parentage assignment and extra–group paternity in a cooperative breeder: the Seychelles warbler (Acrocephalus sechellensis). *Mol. Ecol.*, 10, 2263–2273.

Whiteside, M.A., Horik, J.O. Van, Langley, E.J.G., Beardsworth, C.E., Laker, P.R. & Madden, J.R. (2017). Differences in social preference between the sexes during ontogeny drive segregation in a precocial species. *Behav. Ecol. Sociobiol.*, 71(7), 103.

Woehler, E.E. & Gates, J.M. (1970). An improved method of sexing ring-necked pheasant chicks. *J. Wildl. Manage.*, 34, 228e231.
